# Simultaneous occurrence of bilateral retroperitoneal neuroblastoma and bifocal malignant mixed germ cell tumor in a pediatric patient with 16p11.2 microdeletion syndrome: a case report

**DOI:** 10.3389/fendo.2025.1712251

**Published:** 2026-01-13

**Authors:** Qian-Xiu Fan, Xia-Mei Zhuang, Rong Wen, Xing-Han Wu, Hai-Yan Luo, Hai-Xia Yang, Wen-Yong Kuang, Ben-Shan Zhang, Min-Cui Zheng, Pan Wu

**Affiliations:** 1 Department of Oncology, The Affiliated Children’s Hospital of Xiangya School of Medicine, Central South University (Hunan Children’s Hospital), Changsha, Hunan, China; 2 Department of Radiography, The Affiliated Children’s Hospital of Xiangya School of Medicine, Central South University (Hunan Children’s Hospital), Changsha, Hunan, China; 3 Department of Pathology, The Affiliated Children’s Hospital of Xiangya School of Medicine, Central South University (Hunan Children’s Hospital), Changsha, Hunan, China; 4 Department of Medical Genetics, The Affiliated Children’s Hospital of Xiangya School of Medicine, Central South University (Hunan Children’s Hospital), Changsha, Hunan, China; 5 Department of Hematology, The Affiliated Children’s Hospital of Xiangya School of Medicine, Central South University (Hunan Children’s Hospital), Changsha, Hunan, China

**Keywords:** 16p11.2 microdeletion syndrome, bifocal malignant mixed germ cell tumor, child, coexistence, neuroblastoma

## Abstract

Central nervous system germ cell tumors are rare intracranial neoplasms that predominantly occur in pediatric populations and exhibit characteristics similar to those of gonadal and extragonadal germ cell tumors. Neuroblastoma (NB) represents the most common type of extracranial solid tumor in children, typically arising in tissues with sympathetic innervation. We present a case involving a 14-year-old male patient diagnosed with bilateral intracranial mixed germ cell tumors and concurrent bilateral retroperitoneal ganglioneuroblastoma. To the best of our knowledge, this is the first documented instance of the co-occurrence of these two distinct neoplastic entities. Additionally, Whole-exome sequencing (WES) of the blood sample identified a chromosomal deletion consistent with the 16p11.2 microdeletion syndrome. Furthermore, a heterozygous missense variant in the ALK gene (p. Arg1275Gln) was identified.

## 1 Introduction

Central nervous system germ cell tumors constitute a relatively infrequent category of intracranial neoplasms, predominantly affecting younger individuals. In the United States, the incidence rate is 0.23 per 100,000 individuals. In contrast, the incidence rate of these tumors within Asian nations varies between 0.45 and 0.78 per 100,000 individuals (1–4). According to the 2016 guidelines published by the World Health Organization, central nervous system germ cell tumors are classified into seven distinct types, with mixed germ cell tumors being a significant constituent (5). Neuroblastoma represents the most prevalent form of extracranial solid malignancy among pediatric populations. It originates from progenitor cells located within the neural crest during embryogenesis and has the capacity to emerge throughout the sympathetic nervous system, exhibiting a particular propensity for the adrenal glands (6, 7). The 16p11.2 deletions represent one of the most prevalent causes of neurodevelopmental disorders and autism spectrum disorder, with an occurrence rate of approximately 1 in 2000. Individuals possessing these deletions frequently exhibit a spectrum of clinical manifestations, including delays in early neurodevelopmental milestones. They often experience specific deficits in speech and phonology, language proficiency, diminished cognitive abilities, challenges with motor coordination, and are prone to autism, seizures, and obesity (8).

Previous oncology studies have observed this deletion in germinoma (4), acute myeloid leukemia (9), and neuroblastoma (10), hinting at an oncogenic context. MAPK3 is located within the chromosomal region 16p11.2 and encodes extracellular signal-regulated kinase 1 (ERK1). In a mouse model of autism, the 16p11.2 microdeletion disrupts the balance between neural progenitor cell proliferation and differentiation through dysregulation of the ERK–MAPK signaling cascade, resulting in aberrant brain architecture and behavioral impairments (11). However, the impact on other cancers remains unknown.

In this report, we documented a rare instance of a pediatric patient with 16p11.2 microdeletion syndrome presenting with the simultaneous occurrence of bilateral retroperitoneal neuroblastoma and bifocal malignant mixed germ cell tumor.

## 2 Case presentation

A 14-year-old boy was admitted to the local hospital with a sudden headache, and vomiting and weakness of limbs for 3 hours. Cranial magnetic resonance imaging (MRI) examination of the head showed that the pineal region tumor was complicated with hydrocephalus in the third ventricle, bilateral lateral ventricles, and white matter edema in the ventricle: considering a germ cell tumor, lateral ventricle puncture and external drainage were performed under general anesthesia. Cranial computed tomography showed an epidural hematoma on the right forehead. The patient received brain hematoma removal, other cranial nerve decompression, and skull repair. The family members of the children requested to be transferred to a higher-level hospital for further diagnosis and treatment, so they were transferred to our hospital by ambulance. Physical examination can find language and learning impairment, intellectual disability, macrocephaly, dysmorphic facial features, including forehead protrusion, strabismus, wide mouth, and wide nose. There was no hepatosplenomegaly or lymphadenopathy. The child has a documented history of delayed language acquisition, intellectual impairment, and academic performance challenges. Both parents and the two older sisters have no reported medical conditions and are currently in good health.

**Figure 1:**
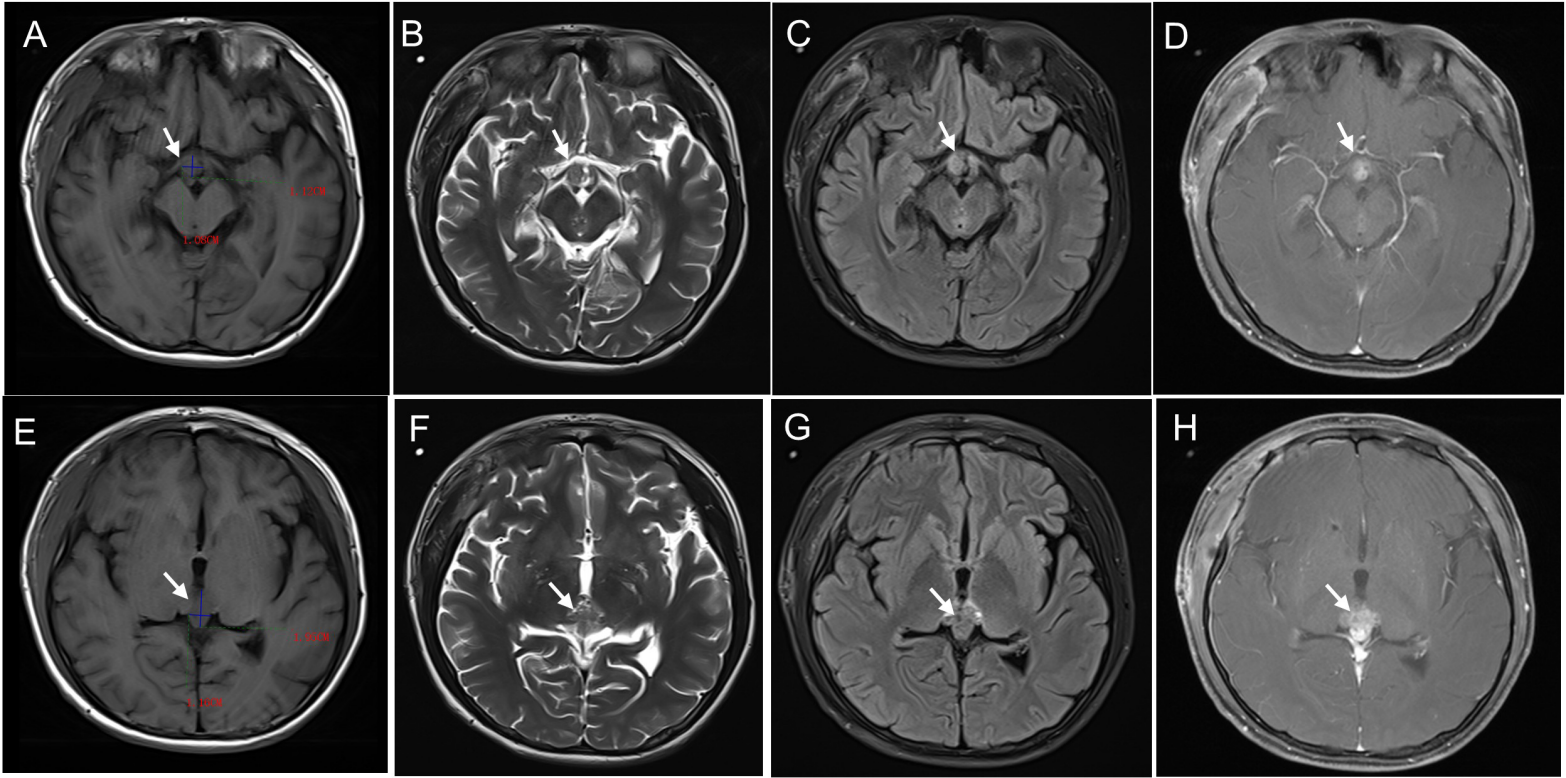
Cranial MRI manifestations of a mixed germ cell tumor in the suprasellar region **(A-D)** and pineal region **(E-F)** . On T1-weighted imaging sequences, the tumor displayed iso- to slightly hypointense signals **(A, E)**. On T2-weighted imaging **(B, F)** and fluid-attenuated inversion recovery (FLAIR) sequences, it exhibited iso- to slightly hyperintense signals. Heterogeneous enhancement was observed on post-contrast images **(D, H)**.

**Figure 2:**
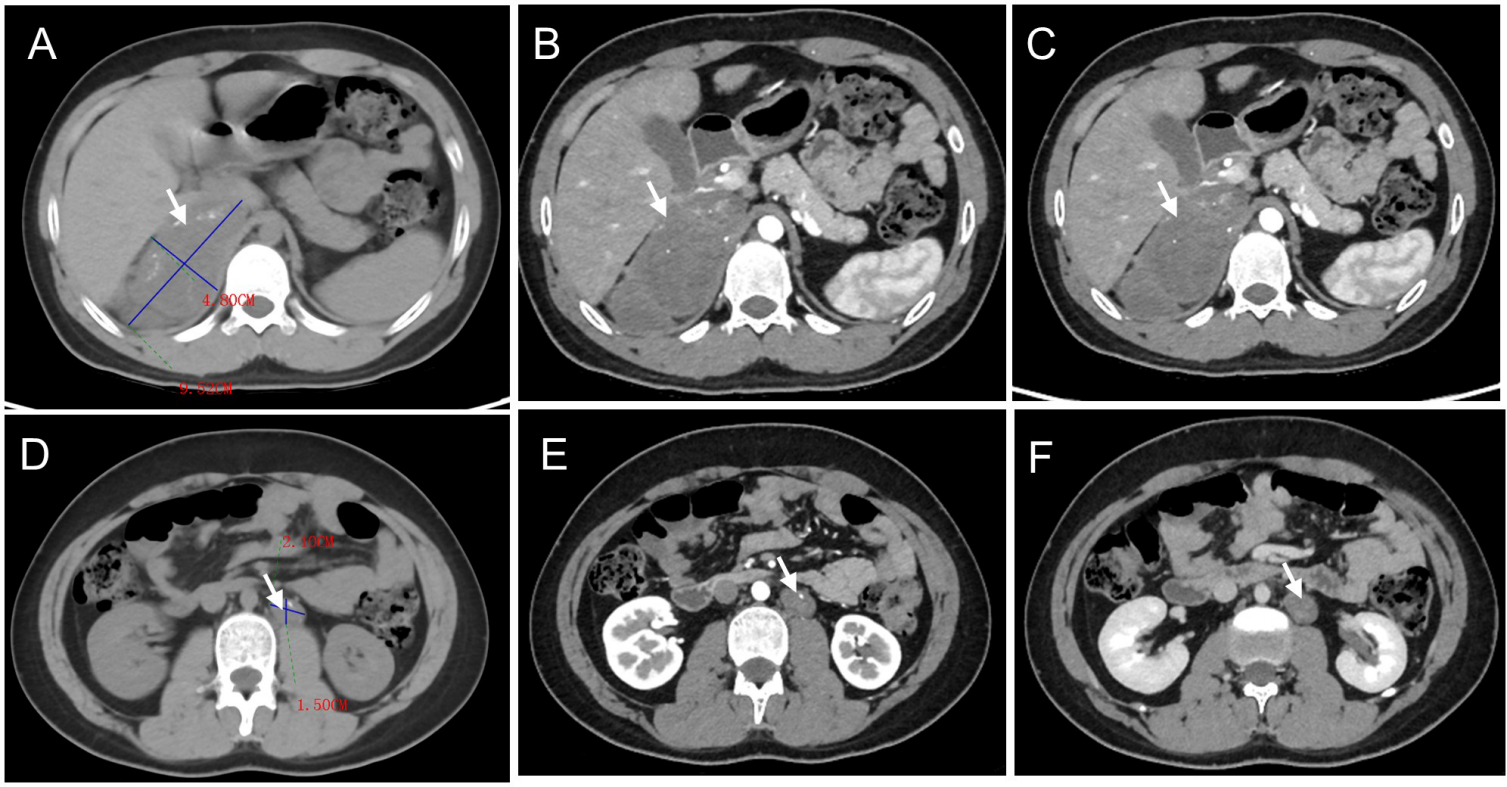
Abdominal CT findings of ganglioneuroblastoma in the same pediatric patient as depicted in **Figure 1**, involving the right **(A-C**) and left **(D-F)** retroperitoneal regions. On non-contrast CT scans (**(A, D)**; lesions indicated by arrows), heterogeneous soft-tissue density masses were identified in the retroperitoneal space, with irregular calcifications observed in both cases. Contrast-enhanced CT scans demonstrated mild heterogeneous enhancement of the masses during the arterial phase (**(B, E)**; arrows) and venous phase (**(C, F)**; arrows).

The patient underwent cranial MRI, which revealed abnormal signal foci in the suprasellar cistern and pineal region. Specifically, one focus measured 1.0 × 1.5 × 0.9 cm in the suprasellar cistern, while another measured 1.5 × 1.7 × 2.0 cm in the pineal region, suggestive of reproductive tumors (**Figure 1**). Additionally, the patient underwent abdominothoracic computed tomography. A mass-like shadow with mixed density was observed in the right retroperitoneal (adrenal) region, measuring approximately 9.7 × 4.4 × 6.7 cm. Furthermore, multiple nodular lesions with heterogeneous density were detected in the left retroperitoneal area, with the largest lesion, measuring approximately 2.2 × 1.8 cm, located inferior to the left renal artery (**Figure 2**).

The biochemical endocrine examination showed the following results: follicle-stimulating hormone (FSH) 0.46 mIU/mL (normal range 1.27-19.46 mIU/mL), luteinising hormone (LH) 0.25 mIU/mL (normal range 1.24-8.62 mIU/mL), total testosterone 907.46 ng/dL (normal range 350-1,070 ng/dL), prolactin 55.28 ng/mL (2.64-13.3 ng/mL), thyroid-stimulating hormone (TSH) 4.517µIU/mL (normal range 0.34-5.6 µIU/mL) and free triiodothyronine 4.62 pmol/L (normal range 3.1-7.7pmol/L), free thyroxine (FT4) 11.08pmol/L (normal range 7.3-22.0 pmol/L), under 50µg/day of levothyroxine, CEA 1.78 ng/mL (normal range 0-5.0 ng/mL), His growth hormone (GH) 0.082ng/mL, analytical tests revealed an adrenocorticotropic hormone (ACTH) plasmatic level at 8AM of 19.3pg/mL, and a cortisol plasmatic level at 0AM of 10.23µg/dL, 8AM of 13.40µg/dL, 4PM 6.92µg/dL (normal range 3.35–11.3µg/dL), Tumor markers alpha-fetoprotein was not elevated in the serum and cerebrospinal fluid, the serum β-subunit of human chorionic gonadotropin (β-hCG) 121.75 mIU/mL (normal range 0–5 mIU/mL), β-hCG in cerebrospinal fluid was significantly elevated in the initial work-up of 430.31 mIU/mL, neuron-specific enolase was normal, The key laboratory parameters were presented in **Table 1**.

**Table 1:** Key laboratory parameters evaluation.

Parameter	Result	Reference range
FSH ( mIU/mL)	0.46	1.27-19.46
LH (mIU/mL)	0.25	1.24-8.62
TT (ng/dL)	907.46	350-1070
Prolactin (ng/mL)	55.28	2.64-13.3
TSH (µIU/mL)	4.517	0.34-5.6
FT3 (pmol/L)	4.62	3.1-7.7
FT4 (pmol/L)	11.08	7.3-22.0
GH (ng/mL)	0.082	0.033-2.47
ACTH 8AM (pg/mL)	19.3	7.2-63.6
Cortisol 0 AM (µg/dL)	10.23	6.7-22.6
Cortisol 8 AM (µg/dL)	13.40	6.7-22.6
Cortisol 4PM (µg/dL)	6.92	3.35–11.3
CEA ( ng/mL)	1.78	0-5.0
Plasma		
AFP (ng/mL)	7.31	0-9
β-hCG ( mIU/mL)	121.75	0-5
cerebrospinal fluid		
AFP (ng/mL)	0.88	NA
β-hCG ( mIU/mL)	430.31	NA
NSE (ng/mL)	13.55	0-16.5

FSH, follicle-stimulating hormone; LH, luteinising hormone; TT, total testosterone; TSH, thyroid-stimulating hormone; FT3, free triiodothyronine; FT4, free thyroxine; GH, growth hormone; ACTH, adrenocorticotropic hormone; CEA, carcinoembryonic antigen; AFP, alpha-fetoprotein; β-hCG, β subunit of human chorionic gonadotropin; NSE, neuron-specific enolase; NA, not applicable.

**Figure 3:**
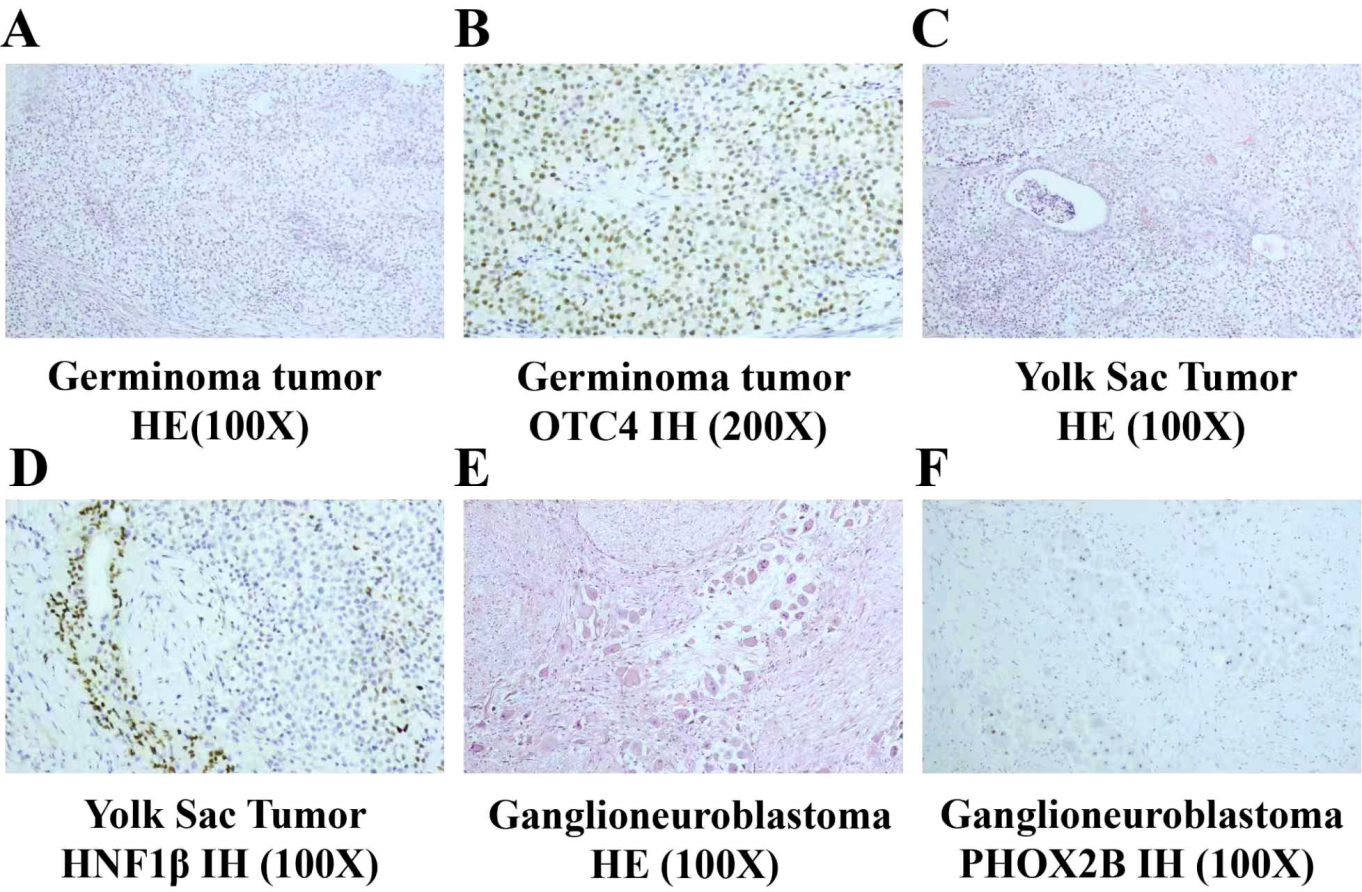
Pathological manifestations of neuroblastoma and malignant Mixed Germ Cell Tumor **(A–F)**. **(A)** Germinoma tumor cells are arranged in diffuse sheets. The tumor cells exhibit clear cytoplasm and large, round nuclei with inconspicuous nucleoli. Lymphocytic infiltration is observed between the tumor cells (×100, HE staining). **(B)** Immunohistochemistry (IH) demonstrates OCT4 nuclear staining positivity (×200). **(C)** A focal component of yolk sac tumor is present. The tumor displays glandular structures (×100, HE staining). **(D)** Immunohistochemistry (IH) for HNF1β reveals nuclear positivity (×200). **(E)** Nests of neuroblasts are arranged in linear arrays within an abundant and well-differentiated Schwannian stroma. The neuroblasts within the nests exhibit good differentiation, with visible neurofibrillary processes (×100). **(F)** Immunohistochemistry (IH) demonstrates PHOX2B nuclear positivity in the neuroblasts.

Considering the possibility that the child harbors tumors of two distinct origins and exhibits symptoms indicative of intracranial hypertension, priority was given to managing the life-threatening intracranial malignant tumor while closely monitoring the abdominal tumor. The patient underwent resection of a space-occupying lesion in the sellar region and resection of a space-occupying lesion in the pineal gland. Following surgery, hydrocortisone-based hormone replacement therapy was initiated for adrenal insufficiency management, desmopressin tablets were administered to control urine volume, and levothyroxine sodium tablets were prescribed to modulate thyroid hormone levels. Pathological immunohistochemical analysis revealed Ki-67 positivity (hotspot area 70%+), Syn (−), CD99 (+), CD34 (vascular and stromal +), S-100 (−), SALL4 (+), OCT-4 (+), INI1 (partial +), Vim (+), CK (+), Glypican (focal dot-like +), AFP (partial +), PLAP (+), CD117 (+), CD30 (−), and α-inhibin (partial weak +), findings consistent with a malignant mixed germ cell tumor. Germ cell tumor components accounted for approximately 97% of the tumors at both sites, while yolk sac tumor components constituted approximately 3% (**Figure 3**). The patient received chemotherapy, alternating between the CE regimen (etoposide 150 mg/m² for 3 days on days 1–3, carboplatin 600 mg/m² on day 1) and the IE regimen (ifosfamide 1.8 g/m² for 5 days on days 1–5, etoposide 100 mg/m² for 5 days on days 1–5) in the treatment protocol.

The abdominal tumor foci were closely monitored during the initial treatment phase and demonstrated no significant changes in size. Following three cycles of chemotherapy, the patient underwent complete surgical resection of bilateral retroperitoneal masses. Pathological examination was consistent with a diagnosis of ganglioneuroblastoma, mixed type. Immunohistochemical analysis revealed Ki-67 (1%+), Chromogranin A (+), Synaptophysin (+), S-100 (+), Neuron-Specific Enolase (+), Protein Gene Product 9.5 (+), PHOX2B (+), and Nestin (+) (**Figure 3**). No evidence of metastasis was observed in other sites. Tissue-specific gene expression analysis confirmed the absence of N-MYC gene amplification, 1p36 gene deletion, and 11q23 gene deletion. Bone marrow aspiration and minimal residual disease (MRD) analysis were negative. The patient was diagnosed with Neuroblastoma (NB), classified as Stage L1 within the Very Low-Risk Group, in accordance with the International Neuroblastoma Risk Group Staging System (INRGSS) (12). After evaluation, it was determined that ganglioneuroblastoma does not require additional chemotherapy and can be managed effectively through close surveillance.

Treatment for intracranial germ cell tumors will proceed as planned with a combination of radiotherapy and chemotherapy. After completing four cycles of chemotherapy, the child underwent fractionated stage-based radiotherapy. First-stage: The planning target volume (PTV) for the whole ventricles and sellar region received a total dose of 36 Gy, delivered in 20 fractions at 1.8 Gy per fraction over 27 days. The planning gross tumor volume (PGTV) for the sellar region and pineal tumor bed received the same total dose regime. Second-stage: The PGTV for the sellar region and pineal tumor bed had received a boost dose of 10 Gy, delivered in 5 fractions at 2.0 Gy per fraction over 7 days. Following radiotherapy, chemotherapy was resumed, resulting in a total of six chemotherapy cycles.

**Figure 4:**
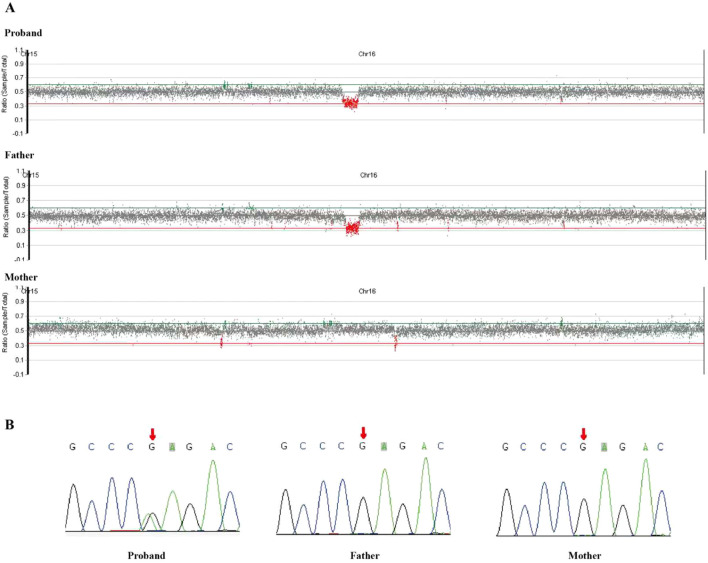
**(A)** WES identified a heterozygous deletion of 562.08 kilobases on the short arm of chromosome 16 [arr[hg19] 16p11.2 (29 675 050–30 237 124) x1]. This deletion, characteristic of the 16p11.2 microdeletion syndrome, was inherited from the patient's father. **(B)** Sanger sequencing of blood samples from the patient's parents was carried out for family verification: A heterozygous missense variant in the ALK gene (NM_004304.5: c.3824G>A, p. Arg1275Gln) was identified in the blood samples. This variant was not detected in the peripheral blood of his parents, suggesting a de novo origin. .

Given the child’s distinctive facial features, developmental delay, and the presence of two malignancies from distinct origins, whole-exome sequencing (WES) was performed for more detailed analysis. WES revealed a heterozygous deletion of 562.08 kilobases on the short arm of chromosome 16 [arr[hg19] 16p11.2 (29 675 050–30 237 124) x1]. This finding is characteristic of the 16p11.2 microdeletion syndrome and was inherited from the father (**Figure 4A**). In addition, a heterozygous missense variant in the ALK gene (NM_004304.5: c.3824G>A, p. Arg1275Gln) was identified in the child’s blood. Notably, this variant was absent in the parents’ peripheral blood samples, indicating a *de novo* origin (**Figure 4B**). The ALK gene has been linked to autosomal dominant or somatic variants that confer susceptibility to neuroblastoma type 3.

**Figure 5:**
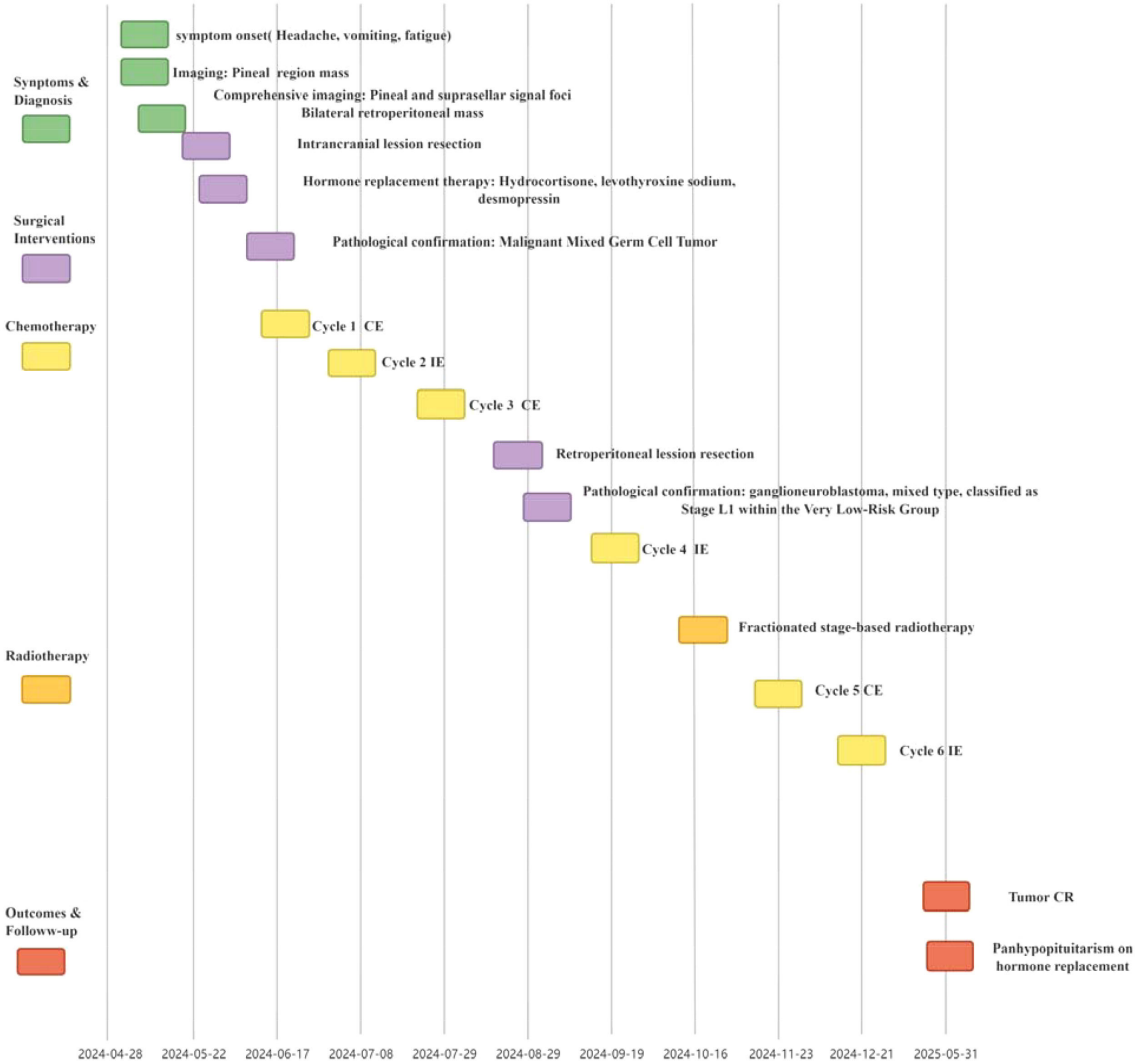
Clinical timeline of the patient. CE, Carboplatin, Etoposide; IE, Ifosfamide, Etoposide; CR, Complete Response.

Upon completion of all chemotherapy sessions, patients were recommended to undergo regular follow-up examinations to monitor treatment response and identify any potential recurrence or late effects. Five months post-chemotherapy, follow-up was conducted through May 2025. The patient was still alive, and two tumors had achieved complete remission. Endocrine evaluation revealed hypopituitarism, and the patient continued to receive levothyroxine and human chorionic gonadotropin (hCG) for hormone replacement. Growth hormone therapy was not initiated due to the family’s concerns regarding potential tumor recurrence. The patient’s clinical course, encompassing initial presentation, treatment, and follow-up, is summarized in **Figure 5**.

## 3 Discussion

Central nervous system germ cell tumors are infrequent neoplasms among children and young adults. They are predominantly found in midline locations, particularly within the pineal and suprasellar regions (13). Approximately 20% of patients may exhibit bifocal pathologies, which simultaneously affect the pineal and suprasellar regions (14). Neuroblastoma is the most prevalent extracranial solid malignant tumor. The most frequent primary sites are the adrenal glands, celiac ganglia, as well as the superior cervical and paraspinal regions (7). Neuroblastoma has been well-documented to coexist with various malignancies, with numerous case reports highlighting its association with Wilms’ tumor. Published cases include: a 4-year-old female with synchronous Wilms tumor and ganglioneuroma (15); a 2-year-old neurofibromatosis patient presenting with Wilms tumor and ganglioneuroblastoma (16); and an infant with Fanconi anemia demonstrating concurrent neuroblastoma and nephroblastoma, Further reports describe an 11-month-old female with VACTERL syndrome and Fanconi anemia exhibiting simultaneous neuroblastoma and nephroblastoma (17), along with other cases involving infants bearing multiple severe congenital anomalies paired with synchronous neuroblastoma and nephroblastoma (18). A particularly notable case documents a patient with Wilms tumor in the right kidney and neuroblastoma in the contralateral kidney (19).

While both Wilms tumor and neuroblastoma predominantly occur sporadically, familial predisposition patterns for these malignancies have been extensively characterized in the clinical literature (20). Beyond Wilms tumor, neuroblastoma has been reported to synchronously manifest with diverse malignant neoplasms, including T-lymphoblastic lymphoma (21), Hepatoblastoma (22), Adrenocortical carcinoma (23), Clear cell sarcoma of the kidney (24), Rhabdomyosarcoma (25) and Langerhans cell histiocytosis (26). Nevertheless, no documented cases of neuroblastoma co-occurring with germ cell tumors have been reported to date. Moreover, the pathophysiological mechanisms underlying the simultaneous development of multiple tumors in pediatric patients remain largely enigmatic.

In the present case, the patient exhibited elevated human chorionic gonadotropin (hCG) in both serum and cerebrospinal fluid. It is important to clarify that exhaustive histopathological examination of the intracranial tumor revealed a mixed germ cell tumor consisting of approximately 97% germinoma and 3% yolk sac tumor, with no distinct trophoblastic or choriocarcinomatous component identified. This observation aligns with contemporary molecular evidence (27), indicating that hCG expression is an inherent capacity of germinoma cells, This expression may originate from scattered syncytiotrophoblastic giant cells or other mechanisms. Furthermore, hCGβ expression is not exclusive to choriocarcinoma but is also detected across other subtypes, including yolk sac tumors.

Germline microdeletions at 16p11.2 are known to predispose individuals to neuroblastoma, as a 550-kilobase deletion within this region is significantly overrepresented among neuroblastoma cases (10). Furthermore, these copy number variants (CNVs) correspond to a well-established microdeletion syndrome associated with an increased risk for various developmental phenotypes, including autism spectrum disorder and other neurodevelopmental disorders (8). Clinical case reports suggest an association between 16p11.2 microdeletion syndrome and the development of certain malignancies, such as acute myeloid leukemia, Endometrial cancer (28), Bifocal germinoma (4), and Wilms’ tumor (29). However, the underlying mechanisms linking 16p11.2 microdeletion syndrome to the development of these malignancies remain poorly understood.

The occurrence of two distinct primary malignant neoplasms in children represents an exceptionally rare clinical scenario. This article presents a novel case of concurrent neuroblastoma and malignant mixed germ cell tumor in a pediatric patient with 16p11.2 Microdeletion Syndrome- a previously unreported combination in medical literature.

The diagnosis of NB at age 14 in this patient is notably rare, as this tumor typically presents in early childhood (median age <2 years) (30). While uncommon, adolescent-onset NB is documented and may be associated with distinct biological behaviors (31). In the context of a genetic predisposition syndrome such as 16p11.2 microdeletion, this delayed clinical manifestation suggests that the microdeletion alone is insufficient for tumorigenesis. Instead, it likely requires a prolonged timeframe for the accumulation of cooperating somatic genetic and epigenetic events to drive clinical disease.

Mitogen-activated protein kinase 3 (MAPK3), located within the 16p11.2 chromosomal region, encodes extracellular signal-regulated kinase 1 (ERK1), which plays a central role in cell proliferation by promoting positive regulators of the cell cycle (32). *In vitro* studies have demonstrated that ERK1 plays a crucial role in regulating differentiation and proliferation in myeloid cells (33). It is therefore plausible that the co-occurrence of malignancies in patients with 16p11.2 microdeletion syndrome may be associated with dysregulation of ERK1-mediated control of cell proliferation. The activity of the MAPK signaling pathway is governed not only by the presence or absence of extracellular stimuli, but also by the intensity, duration, and oscillatory dynamics of the signal (34). This may contribute to the delayed onset of tumorigenesis. The delayed manifestation of tumors underscores the protracted nature of this multi-step, multifactorial accumulation process. Confirming this association and translating it into effective long-term health management for carriers will require future longitudinal cohort studies and in-depth mechanistic investigations.

Copy number aberrations involving the ALK gene are frequent genetic events in the development of neuroblastoma. ALK contributes to neuroblastoma pathogenesis, in part, through a feedforward loop between POSTN and WNT signaling (35, 36). The presence of a heterozygous missense variant in the ALK gene in this patient may represent a second hit, thereby contributing to the development of neuroblastoma.

During follow-up, the child was diagnosed with hypopituitarism and consequently required hormone replacement therapy. In patients with intracranial tumors—particularly those with lesions in the sellar or pineal regions—multimodal treatment, including chemotherapy, surgery, and radiotherapy, is commonly necessary. Both the disease itself and its treatment may compromise the hypothalamic-pituitary axis, underscoring the importance of close endocrine monitoring and timely initiation of hormone replacement therapy, which plays a critical role in improving long-term outcomes in pediatric patients.

In summary, this case has provided valuable clinical insights and practical experience. First, in children presenting with anatomically distinct tumor foci, diagnostic considerations must extend beyond metastasis to include synchronous primary malignancies. Second, when dual primary tumors are suspected, the therapeutic focus should immediately address the life-threatening lesion while instituting rigorous surveillance for the secondary tumor. Third, Histopathological biopsy constitutes the definitive method for distinguishing tumor origins. Poor therapeutic response in any lesion warrants surgical resection for diagnostic clarification. Finally, comprehensive germline testing is essential in synchronous tumor presentations, given the potential association with tumor predisposition syndromes.

Patient Perspective: Written informed consent was obtained from the patient’s legal guardian for the publication of this case report and any accompanying images. The guardian has reviewed the final manuscript and confirms that anonymized data may be used for scientific and educational purposes.

## Data Availability

The original contributions presented in the study are included in the article material. Further inquiries can be directed to the corresponding author.
